# Delirium in a geriatric patient with Parkinson’s disease, management and outcomes: a case report

**DOI:** 10.1192/j.eurpsy.2025.1727

**Published:** 2025-08-26

**Authors:** I. Contreras Pérez, A. Jurado Arévalo, M. P. Vargas Melero, I. Caparrós del Moral, R. Carrillo Molina, F. Vílchez Español

**Affiliations:** 1 Psiquiatría, Hospital Neurotraumatológico, Jaén, Spain

## Abstract

**Introduction:**

Up to 40% of patients diagnosed with Parkinson’s disease (PD) may experience a psychotic episode during the course of the disease, with antiparkinsonian medications being the main cause. Frequently, aging is associated with a higher risk of comorbid delirium in this population.

**Objectives:**

To analyze the treatment strategy for delirium in a geriatric patient with Parkinson’s disease.

**Methods:**

An 88-year-old male patient, diagnosed with Parkinson’s disease for 12 years, was admitted to the Acute Psychiatry Hospitalization Unit due to a treatment-resistant confusional state. He presented fluctuating symptoms characterized by verbal and physical hetero-aggressiveness, visual illusions and hallucinations, as well as delusional ideas of harm, control, and mystical-religious content. The patient exhibited significant psychological distress, refused to eat, and had erratic medication adherence.

**Results:**

Following a comprehensive organic assessment and treatment of intercurrent conditions, a readjustment of dopaminergic medication was performed, and quetiapine was introduced (up to 900 mg/day), with a partial response. Subsequently, the doses of quetiapine were reduced, and ziprasidone was introduced, achieving total remission of symptoms with good tolerance (quetiapine 450 mg/day, ziprasidone 80 mg/day, levodopa/carbidopa 150 mg/day).

**Conclusions:**

Following pharmacological recommendations for managing delirium, initial treatment with quetiapine (first-line) was established. Subsequently, clozapine (second-line) was introduced, achieving a better response and cessation of symptoms. This case highlights the complexity of managing delirium in geriatric patients with PD, considering the patient’s age and lack of response to standard therapeutic guidelines. Clozapine, with a more favorable profile regarding motor effects, presents as the preferred option compared to other antipsychotics that may exacerbate parkinsonism.

**Disclosure of Interest:**

None Declared

